# *PAL1* gene of the phenylpropanoid pathway increases resistance to the *Cassava brown streak virus* in cassava

**DOI:** 10.1186/s12985-021-01649-2

**Published:** 2021-09-09

**Authors:** Siji Kavil, Gerald Otti, Sophie Bouvaine, Andrew Armitage, Midatharahally N. Maruthi

**Affiliations:** 1 Agriculture, Health and Environment Department, Natural Resources Institute, University of Greenwich, Medway campus, Chatham, Kent ME4 4TB UK; 2 Computational and Analytical Sciences, Rothamsted Research, Harpenden, AL5 2JQ UK

**Keywords:** Cassava, CBSD, *PAL1*, Resistance, Induction

## Abstract

**Background:**

The phenylalanine ammonia lyase genes play crucial role in plant response to biotic and abiotic stresses. In this study, we characterized the role of PAL genes in increasing resistance to the *Cassava brown streak virus* that causes the economically important cassava brown streak disease (CBSD) on cassava in Africa.

**Methods:**

The whole transcriptomes of eight cassava varieties differing in resistance to CBSD were obtained at 1, 5 and 8 weeks after CBSV infection.

**Results:**

Analysis of RNA-Seq data identified the overexpression of *PAL1, PAL2*, cinnamic acid and two chalcone synthase genes in CBSD-resistant cassava varieties, which was subsequently confirmed by RT-qPCR. The exogenous application of Acibenzolar-S-Methyl induced *PAL1* gene expression to enhance resistance in the susceptible var. Kalawe. In contrast, the silencing of *PAL1* by RNA interference led to increased susceptibility of the resistant var. Kaleso to CBSD.

**Conclusions:**

*PAL1* gene of the phenylpropanoid pathway has a major role in inducing resistance to CBSD in cassava plants and its early induction is key for CBSD resistance.

**Supplementary Information:**

The online version contains supplementary material available at 10.1186/s12985-021-01649-2.

## Introduction

**Fig. 1:**
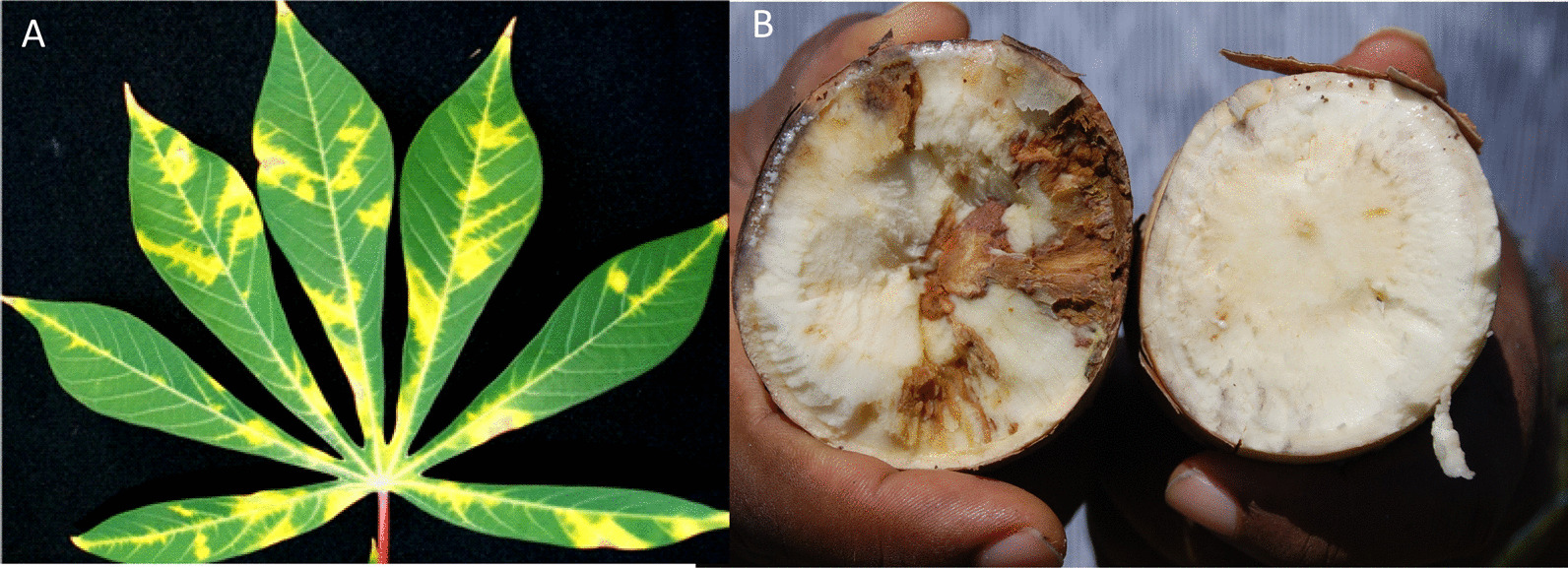
**A** Typical chlorotic symptoms of CBSD on cassava leaves, and **B** dry necrotic rotting of infected cassava root (left) compared to the healthy root (right)

Cassava is an important food crop for > 450 million people in Africa but, suffers significant production losses caused by the highly damaging cassava brown streak disease (CBSD). CBSD is caused by two RNA virus species; *Cassava brown streak virus* (CBSV) and *Ugandan cassava brown streak virus* (UCBSV) [1, 2] together known as cassava brown streak ipomoviruses (CBSIs) [3]. The characteristic symptoms of CBSD include severe chlorosis on infected leaves (Fig. 1a), and necrosis and dry rotting of roots in susceptible varieties (Fig. 1b), which makes the roots unfit for consumption or marketing. CBSD is the greatest threat to food security and, it is a problem to communities in eastern, central and parts of southern Africa that rely on cassava [4–7]. The disease causes losses in excess of US$750 million annually [6]. Recent efforts in Eastern Africa have, identified several cassava varieties resistant/ tolerant to the disease, which show no, mild or delayed symptoms of root necrosis upon CBSV infection [8–10]. Attempts have been made to understand the mechanism of CBSD resistance by transcriptome profiling (RNA-Seq) of CBSD-resistant and -susceptible cassava varieties [8, 11, 12]. These studies provided insights into the genes modulated by CBSV infection, however, no specific genes were directly implicated in resistance and thus the mechanism of resistance is poorly understood. Developing gene-targeted molecular markers for breeding can significantly contribute to sustainable control of the disease [1, 11, 12].

Plants have developed a multi-layered defense network to detect invading pathogens and stop them before they cause extensive damage. Phenylpropanoid pathway is important in plant’s defense mechanisms and provides structural and chemical barriers for resistance to pathogen infection. During pathogen attack, phenylpropanoid pathway genes were found overexpressed, resulting in increased enzymatic activities and accumulation of various phenolic compounds [13–15]. Marked increase in phenylalanine ammonia lyase (PAL) gene expression has been observed in many plant-pathogen interactions in response to microbial or endogenous elicitors [16, 17]. PAL is therefore considered a chemical marker of induced resistance in many plants. Expression of PAL could be manipulated to improve disease resistance in plants. In vitro use of elicitors on plants can activate various biosynthetic pathways such as jasmonic acid (JA), salicylic acid (SA), ethylene and phenylpropanoid pathway, similar to the induction by the pathogen infection [18, 19]. A functional analogue of salicylic acid, acibenzolar-S-Methyl (ASM), is one of the well-known inducers of systemic acquired resistance (SAR) for numerous plant species [20–22], but not investigated in cassava previously. In this study, we investigated the role of *PAL1* in CBSD resistance upon induction by ASM as well as by suppression through RNA interference (RNAi). Here, we characterized the transcriptomic response of CBSD-resistant, tolerant, and susceptible cassava varieties at different times after CBSV infection to identify difference in their defence response.

## Methods

### Cassava varieties and virus inoculation for transcriptome analyses

Eight cassava varieties differing in resistance levels to CBSD (resistant, tolerant, and susceptible) were used in a transcriptome time series experiment (Table 1). Cassava plants were grown from cuttings at 28 ± 5 °C with 50–60% relative humidity for 2 months. Plants of var. Albert infected with CBSV isolate (MZ:Nam1-1:07) [23] were used as virus source to inoculate eight, two months old cassava plants by side-wedge grafting method [24]. In each variety, a maximum of five plants were graft-inoculated with the virus alongside an additional three plants grafted with a healthy scion (mock inoculation control). Leaf samples were collected from infected and control plants at 1, 5 and 8 weeks after inoculation (wai) by clipping a single lobe from one of the leaves in the top, middle and lower parts of each plant. Samples were immediately frozen in liquid nitrogen and either processed immediately or stored at -80 °C. Equal amounts of leaf tissue were pooled from three replicate plants of the same variety prior to RNA extraction.

**Table 1:** Cassava varieties subjected to RNA sequencing and transcriptome analysis

Cassava variety	CBSD phenotype*
Kaleso	Resistant
Mkumba	Resistant
Pwani	Resistant
Nase 3	Resistant
Oekhumelela	Tolerant
Kiroba	Tolerant
Albert	Susceptible
Kalawe	Susceptible

*Level of resistance of the cassava varieties based on CBSV accumulation levels in glasshouse conditions [1, 23]

### RNA extraction and Illumina RNA-Seq

RNA was extracted from cassava leaves using an adapted protocol which combined a modified Cetyltrimethylammonium bromide (CTAB) method [25, 26] and the RNA extraction protocol of the Sigma’s Spectrum TM Plant Total RNA Kit. About 100 mg of frozen cassava leaf tissue samples were homogenized using bullet blender (Next advance, USA) in 1 ml of pre-heated CTAB buffer and incubated at 60 °C, for 5 min. Samples were centrifuged and clear lysates were mixed thoroughly with 500 µl of binding solution. Mixture was transferred to RNA kit spin column and centrifuged at 21,129 g and the flow through was discarded. The spin column was dried by additional centrifugation and the column was transferred to fresh 2 ml collection tube. Purified RNA was eluted in 50 µl of elution buffer. RNA purity and quantities were measured using a NanoDrop spectrophotometer (Thermo scientific, Wilmington USA). RNA integrity (RIN) values were determined using an Agilent 2100 Bioanalyzer instrument (Agilent Technologies, CA USA). Messenger RNA (mRNA) library preparation was performed using Illumina’s TruSeq RNA library preparation kit by the Earlham Institute, Norwich, UK. The resulting cDNA libraries were indexed with TruSeq index adapter barcode tags, checked for quality and sequenced in multiplexed mixtures of 6 libraries per lane using Illumina’s HiSeq 2500 next-generation sequencing system.

### Sequence processing, alignment to the cassava genome and identification of differentially expressed genes (DEGs)

Raw RNA-Seq data was trimmed, and adapters removed using FASTA/Q trimmer tool of the FASTX-TOOLKIT collection (http://hannonlab.cshl.edu/fastx_toolkit/commandline.html). Processed reads were mapped to the JGI *Manihot esculenta* v4.1 reference genome (http://phytozome.jgi.doe.gov/pz/portal.html#!info?alias=Org_Mesculenta) using TopHat v.2.1.1 [27]. Mapped reads were quantified at the gene level using the Linux-based tool generalized fold change (GFOLD V1.1.0) in relation to the *Manihot esculenta* v4.1 gene models [28]. Differential gene expression was reported by GFOLD based on the posterior distribution of log2 fold change (LFC) calculated from the gene expression value (RPKM) in CBSV-inoculated sample compared to the corresponding mock-inoculated samples. Genes with GFOLD LFC values > 1 were considered induced and those with < −1 considered repressed. Effects of sampling time, variety grouping and CBSV infection status were determined using Extraction of Differential Gene Expression (edge 2.8.0) package in R software [29].

Gene functional annotations associated with *M. esculenta* v.7.1 gene models were used to investigate DEGs. Additionally, gene identities from earlier *M. esculenta* were associated with v.7.1 gene IDs using publicly available resources (https://phytozome.jgi.doe.gov/pz/portal.html#!info?alias=Org_Mesculenta). Where functional annotations were unavailable, putative function was assigned to DEGs identified through BLAST-searching their amino acid sequences against *Arabidopsis thaliana* genome on the STRING protein network interaction platform (STRING v11) [30]. Assigned putative functions were grouped into functional categories and further checked against cassava genome database at Phytozome portal of JGI (https://phytozome.jgi.doe.gov/pz/portal.html). The net modulation of genes belonging to each functional category was assessed by calculating time-course average in total fold difference (between inoculated and control) for genes of the category and normalized by the average of the total fold for the entire transcriptome. The one minus Pearson’s correlation distance measure was used for hierarchical clustering of expression levels of individual genes. The 48 sequenced cassava samples were grouped according to the three factors—type of inoculation (CBSV- or mock-inoculated), varieties (resistant, tolerant, or susceptible) and post-inoculation sampling time. The maximum number of contiguous samples of a single group which partition into same cluster was used to measure the effect of each sample grouping factors. In addition to the wider analyses, we set out to identify DEGs involved in SA, JA and phenylpropanoid pathway genes up on CBSV infection.

### Phenylpropanoid pathway gene expression

A qPCR-based expression analysis was performed on two PAL genes, two chalcone synthase (CHS) genes and one cinnamic acid (*C4H*) gene in two cassava varieties that contrasted in CBSD resistance (Kaleso and Kalawe). Six months old cassava plants were graft inoculated with CBSV by side-wedge grafting method, leaf samples were collected from CBSV- and mock- inoculated plants at 2, 4 and 14 days after inoculation (dai) for RNA extraction. Total RNA was extracted, stored, and pooled as explained above. The total RNA extracted was treated with DNase I (Invitrogen, California, United States) and 1 g of total RNA was reverse transcribed using ImProm-II Reverse Transcriptase (Promega, UK). qPCR was performed with SYBR Green (Thermo-Fisher, UK) using Bio-Rad CFX96 Real Time System (Bio-Rad laboratories, USA) using gene specific primers (Additional file 1: Table S1). The relative expression levels of individual genes in each sample was calculated using 2^ΔΔCq^ method [31]. For each CBSV inoculated sample, respective control (mock) sample was used as calibrator for gene expression pairwise comparison. Fold expression represented in log2 fold change (LFC).

### Induction of for *PAL1* gene with acibenzolar-S-methyl application

Induction of *PAL1* by ASM was carried out on six months old Kaleso and Kalawe plants by spraying 1.18 mM of ASM twice daily for seven days. Water sprayed plants were used as controls and, six plants were sprayed per variety and treatments. All the plants were inoculated with CBSV by side wedge grafting with an infected scion of var. Albert at 8 h after the first spraying of ASM. A single lobe of a fully expanded leaf (third or fourth leaf from the top) was collected from each plant at 8 h, and at 1, 2, 7, 14, 21 and 28 days after treatment (dat) and used for estimating *PAL1* expression as well as virus quantification.

### RNAi suppression of *PAL1*

Cassava *PAL1* gene sequences were blast searched (BLASTn) in Phytozome and highly conserved regions of 300 bp was selected for silencing (Additional file 1: Table S2). *PAL1* gene was amplified from cassava cDNA using primers with 5′ adaptor (*att*B) sequences and cloned into pHELLSGATE vector (CISRO, Australia) using Gateway cloning to prepare the RNAi construct (Additional file 1: Table S1) [32]. pHELLSGATE-*PAL1* RNAi construct was transformed into competent cells of the super virulent *Agrobacterium* strain AGL1 by freeze–thaw method. The pHELLSGATE-*PAL1* and empty vectors (pHELLSGATE) containing *Agrobacterium* clones were induced and used for agro-inoculation of cassava var. Kaleso by both syringe [33] and prick inoculation methods with toothpicks [34].

Booster inoculations of pHELLSGATE-*PAL1* were given to the plants twice at 20 days intervals from the first inoculation to maintain/extend the effects of gene silencing. All agro-inoculated and control (inoculated with empty vector) Kaleso plants were inoculated with CBSV by side grafting at 20 days after agro-inoculation (daai). CBSD susceptible Kalawe plants were also maintained as control for virus comparison. Leaf samples were collected at 0, 20, 34, 41 and 48 daai, snap-frozen and stored at − 80 °C. RNA extracted from these samples were used to estimate the suppression of *PAL1* gene as well as for virus quantification (Additional file 1: Table S1).

## Results

### Cassava transcriptome analysis

**Fig. 2:**
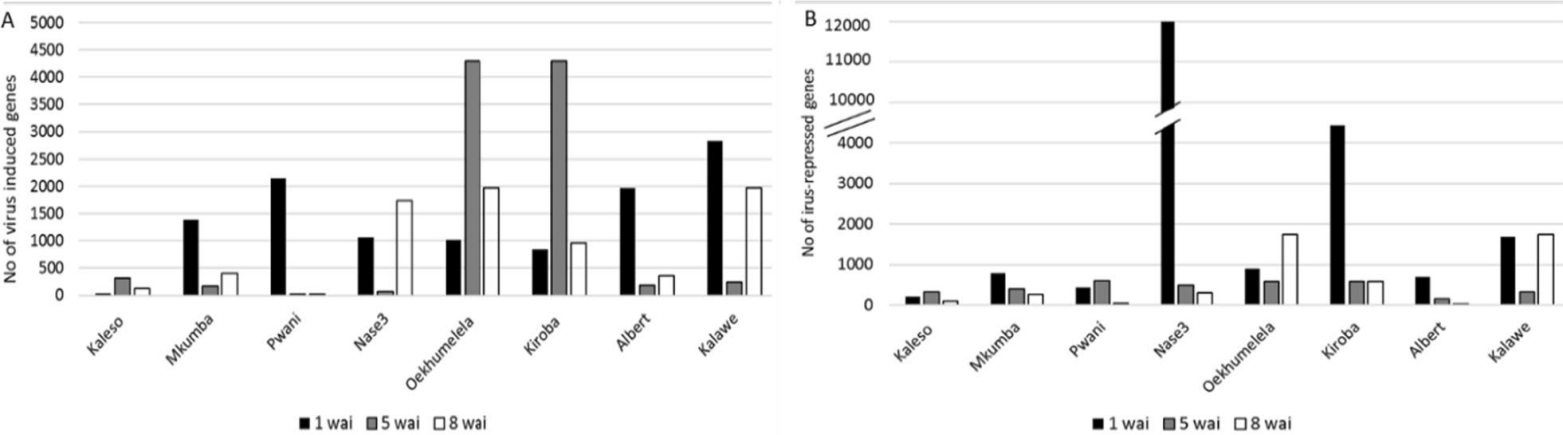
Total number of virus induced (**A**) and virus repressed genes (**B**) identified from eight cassava varieties at 1, 5 and 8 wai. Pairwise comparison of RPKM values between the CBSV-inoculated and control samples identified the virus induced and repressed genes

mRNA sequences (RNA-Seq data) generated from the 48 cassava samples (8 varieties × 2 treatments: CBSV/mock × 3 time points) were mapped to the reference cassava genome (*Manihot esculenta* v4.1). On average 88% of the reads were mapped to *M. esculenta* and CBSV genomes. Comparison of gene expression between virus- and mock-inoculated cassava samples, based on GFOLD analysis, identified a total of 8971 DEGs (virus induced or repressed) across the eight cassava varieties. Highest number of DEGs were found at the earliest time point of CBSV infection, 1 week after inoculation (wai) in all varieties except for the resistant Kaleso which had the highest number of modulated genes (632) at 5 wai (Fig. 2). Among the cassava varieties, the CBSD-tolerant Oekhumelela and Kiroba had the highest number of DEGs (1853) compared to susceptible and resistant varieties. Resistant variety, Kaleso had lowest number of DEGs (366) followed by Mkumba (1131; Fig. 2). Pairwise comparison of RPKM values between the CBSV-inoculated and control samples identified on average 6554 and 9804 virus induced and repressed genes (Fig. 2), respectively.

Sampling time influenced the transcriptome profiles more than the resistance levels of the cassava varieties; resistant, tolerant, or susceptible (Edge analysis). This was also confirmed by hierarchical clustering (Additional file 1: Fig. S1). Among the eight cassava varieties, a total of 13,058 DEGs showed significant modulation (FDR < 0.05) over the three sampling times. In contrast, only 4223 DEGs showed significant modulation between paired combinations of resistant, tolerant, and susceptible varieties. Specifically, 2044, 1976 and 203 DEGs showed significant modulation (FDR < 0.05, Benjamini-Hochberg) between the tolerant and resistant varieties, susceptible and resistant varieties, and tolerant and susceptible varieties (FDR < 0.05, Benjamini-Hochberg), respectively.

### Genes and signalling pathways enriched in resistant varieties

**Fig. 3:**
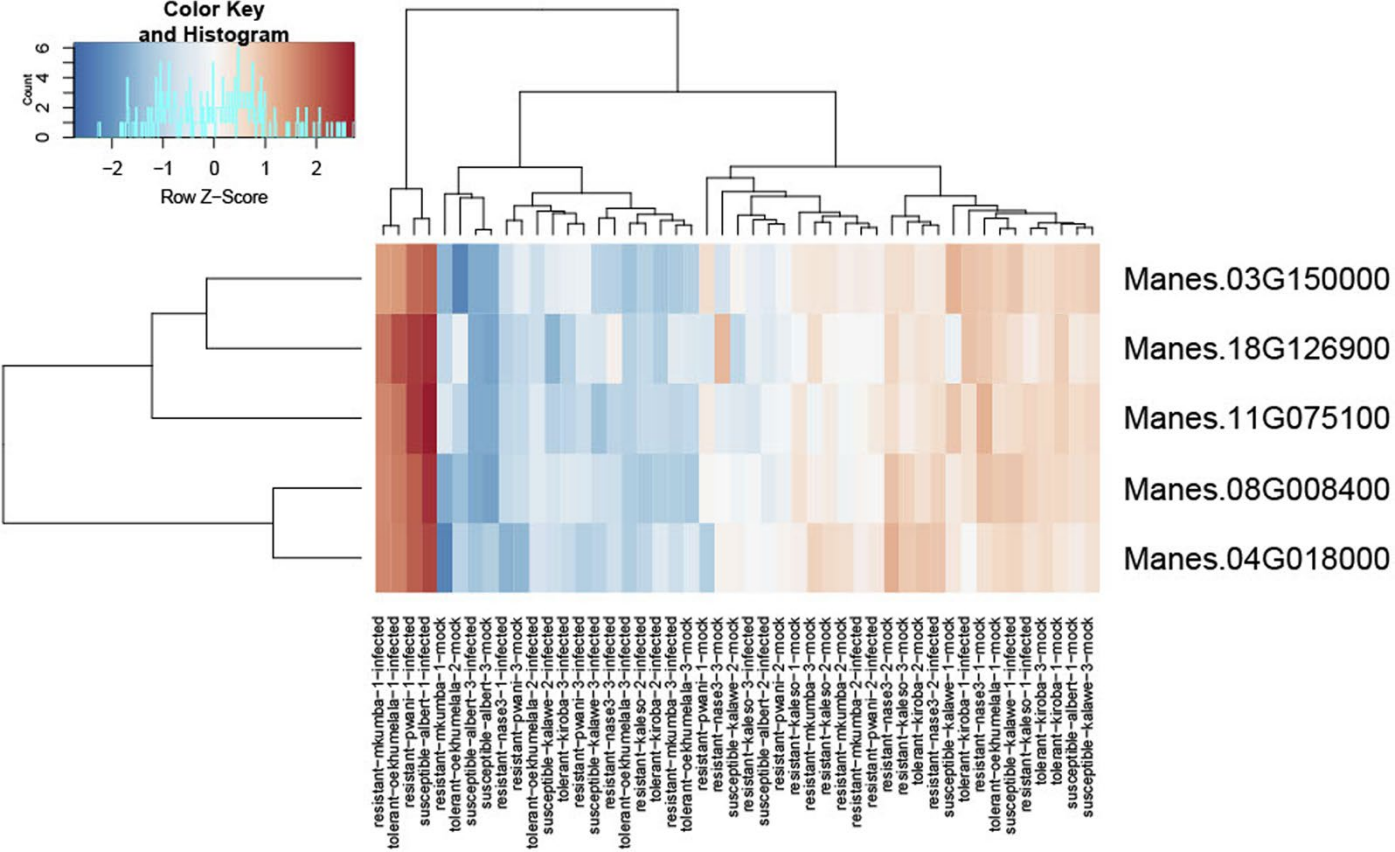
Expression profiles of selected five phenylpropanoid genes at 2, 4 and 14 dai

CBSV also induced genes coding for abiotic stress, antioxidant defense, cell wall loosening/ cell expansion and pathogenesis-related functions in susceptible varieties but repressed in the resistant varieties (Additional file 1: Figs. S2, S3 and S4). Nucleotide binding site-Leucine rich repeat (NBS-LRR) genes were induced in both resistant and susceptible varieties at 1 wai (Additional file 1: Figs. S2 and S4), however, the mean induction of NBS-LRR genes was higher in resistant up to 4.2 log2 fold change (LFC) compared to susceptible varieties. Virus infection also modulated the expression of signaling pathways. Genes involved in JA signaling and biosynthesis pathways were repressed at early stage of CBSV infection (1 wai) in resistant varieties but induced in tolerant and susceptible varieties (Additional file 1: Figs. S2, S3 and S4). In the phenylpropanoid pathway, 52 virus induced genes were identified, and the fold induction varied among the varieties and time points. Mean induction of phenylpropanoid pathway genes was higher (4.3 fold more) in resistant varieties at early time point of CBSV infection at 1 wai compared to tolerant and susceptible varieties (Additional file 1: Figs. S2, S3 and S4). Among the virus induced phenylpropanoid pathway genes, five (*PAL1, PAL2*, one C4H and two CHSs) were overexpressed in resistant/tolerant varieties at early time point (1 wai) compared to susceptible varieties (Fig. 3). Highest induction of *PAL1* and *PAL2* was found in Mkumba (6.5 and 6.4 LFC respectively) followed by Pwani (4.8 and 3.6 LFC respectively) at 1 wai. Expression of these five genes was measured by RT-qPCR on leaf samples from CBSD-resistant Kaleso and susceptible Kalawe, collected in an independent experiment at earlier times after inoculation: 2, 4 and 14 dai. Both the PAL genes (*PAL1* and *PAL2*) showed induction upon virus infection in the resistant Kaleso from 2 dai (Table 2). In the susceptible Kalawe, *PAL1* (Manes.04G018000) showed late induction at 14 dai (3.3 LFC) while *PAL2* (Manes.08G008400) was repressed at all time points (Table 2). Similarly, CHS genes (Manes.11G075100.1 and Manes.03G150000.1) as well as *C4H* (Manes.18G126900.1) showed early induction at 2 dai (2.2, 1.5 and 5.4 LFC respectively) in Kaleso but they were induced late or repressed in Kalawe.

**Table 2:** Relative expression (log_2_ fold change, LFC) of phenylpropanoid pathway genes at 2, 4 and 14 dai

Gene IDs*	Protein name	Variety	LFC at 2–14 dai		
			2	4	14
Manes.04G018000.1	*PAL1*	Kaleso	5.4	4.5	3.3
		Kalawe	− 0.5	− 10.0	5.1
Manes.08G008400.1	*PAL2*	Kaleso	4.9	2.8	0.3
		Kalawe	0.9	− 5.6	− 5.1
Manes.18G126900.1	C4H	Kaleso	5.4	6.3	− 1.0
		Kalawe	0.0	− 3.3	3.6
Manes.11G075100.1	CHS	Kaleso	2.2	2.4	3.8
		Kalawe	0.1	− 4.6	3.7
Manes.03G150000.1	CHS	Kaleso	1.5	2.1	2.8
		Kalawe	− 0.5	− 5.1	2.9

### Over expression of *PAL1* in the susceptible var. Kalawe for CBSD resistance

**Fig. 4:**
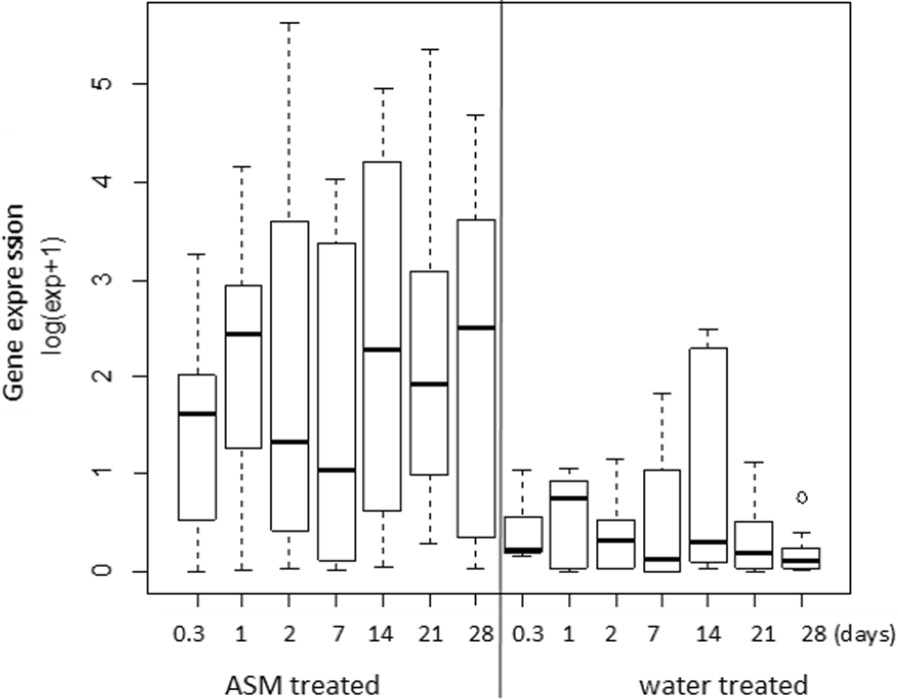
Whisker box plot analysis of *PAL1* expression levels in ASM- and water-treated samples (Kaleso and Kalawe combined) normalized to respective 0 dat, at different time points. Significant difference in *PAL1* gene expression was observed between the ASM- and water-treated plants (p < 0.001), 0.3 day = 8 hat

Effective induction of *PAL1* was noted in both ASM-treated Kaleso and Kalawe plants and the magnitude of induction varied depending on the variety and time of samples collection (Table 3). In Kaleso, *PAL1* was induced in ASM-treated Kaleso plants starting at 8 h after treatment (hat), peaked at 2 dat (7.3 LFC) and remained high until 28 dat. *PAL1* was also induced in ASM-treated CBSV-inoculated Kalawe plants from 8 hat, peaked at 1 dat albeit to lesser extent. *PAL1* was not induced in ASM-treated mock-inoculated Kalawe plants and in control plants (water-treated Kalawe plants with/without CBSV). Virus titer on both ASM- and water-treated control cassava plants were quantified by qPCR at 14, 21 and 28 dai (Table 3). CBSV was not detected in ASM-treated susceptible Kalawe plants until 21 dai while in water-treated control plants, the virus was detected early, by 14 dai. Quantity of CBSV in ASM-treated Kalawe plants was up to 3-times lower at 21 and 28 dai compared to control plants. Statistical analysis showed significant differences in *PAL1* expression between the ASM- and water-treated control samples (F = 113, df = 1, 4, p = 0.00044) (Fig. 4).

**Table 3:** Time course measurement of log_2_ fold change (LFC) in *PAL1* expression in ASM- and water-treated Kaleso and Kalawe plants

Sample type	LFC of *PAL1* in RNAi-silenced plants at 0.3–28 days post agro-infiltration:							LFC of CBSV abundance at 14–28 dai of treated plants:		
	0.3	1	2	7	14	21	28	14	21	28
Kaleso ASM CBSV	2.4	4.8	4.7	4.6	6.3	3.5	5.6	ND	ND	ND
Kaleso ASM Mock	3.7	3.3	7.3	5.3	5.9	7.0	5.8	ND	ND	ND
Kaleso water CBSV	0.7	0.6	1.5	1.5	3.3	0.3	− 0.9	ND	ND	ND
Kaleso water Mock	− 2.6	0.8	0.4	0.4	− 0.9	1.1	− 3.2	ND	ND	ND
Kalawe ASM CBSV	2.0	3.4	0.1	− 1.6	2.6	2.8	2.4	ND	− 10.4	− 15.4
Kalawe ASM Mock	− 6.1	− 6.4	− 5.0	− 5.9	− 2.8	− 1.5	− 1.6	ND	ND	ND
Kalawe water CBSV	− 0.6	− 1.3	− 2.4	− 4.1	− 2.3	− 0.4	− 2.6	− 1.0	− 4.7	1.1
Kalawe water Mock	0.2	− 6.3	− 5.0	− 9.4	− 5.0	− 6.4	− 5.1	ND	ND	ND

Not detectable virus quantities are marked as ND

### Suppression of *PAL1* in the resistant var. Kaleso by RNAi leads to susceptibility

RNAi-mediated gene silencing was used to further confirm the role of *PAL1* gene in CBSD resistance. Relative expression analysis confirmed the repression of *PAL1* (-0.2 to -13.6 LFC) starting 20 days after agro-inoculation (daai) in the silenced Kaleso plants compared to before silencing (0 daai). Plants with consistent repression of *PAL1* became infected with CBSV early, at 21 dai and the virus persisted till 48 daai. In *PAL1* silenced Kaleso plant, the highest virus titer was measured at 21 daai (5.9 LFC) compared to virus-control plants (Kalawe). Control Kaleso plants inoculated with empty vector remained virus free during the experiment (Table 4). Our results indicated that booster inoculation of silencing construct was necessary for maintaining the silencing of *PAL1* gene wherein one of the silenced plants showed *PAL1* reduction only after first booster inoculation, at 34 daai (Table 4).

**Table 4:** RT-qPCR-based relative expression (log_2_ fold change, LFC) of transcripts and CBSV concentration in RNAi-silenced/control Kaleso plants at different time points of agro-inoculation relative to 0 daai

Sample type	LFC of *PAL1* in RNAi-silenced plants at 20–48 days post agro-infiltration:				LFC of CBSV abundance at 14–28 days after inoculation of treated plants:		
	20	34	41	48	34	41	48
Non-silenced, absolute control	0.5	3.7	2.0	4.8	ND	ND	ND
	− 0.2	− 0.7	2.0	3.8	ND	ND	ND
Non-silenced, control with empty vector	− 0.3	1.3	− 3.3	− 0.7	ND	ND	ND
	− 1.0	− 0.7	− 3.3	− 9.0	ND	ND	ND
CBSV inoculation control, susceptible Kalawe	0.6	− 3.3	− 0.5	1.8	0.0	3.7	6.1
	1.0	− 0.7	0.9	1.4	ND	0.9	5.9
	− 1.7	0.8	− 10.0	1.0	ND	ND	0.4
	− 0.2	− 2.3	− 13.3	− 11.3	ND	ND	ND
*PAL1* silenced Kaleso plants	− 3.3	− 7.6	− 3.3	− 7.0	ND	5.9	0.4
	− 8.0	− 1.7	− 0.3	2.0	ND	ND	ND
	1.1	2.5	2.7	3.4	ND	ND	ND

Not detectable virus quantities are marked as ND

## Discussion

CBSD causes an estimated loss of about US $750 million in the eastern African countries, endangering the food and financial securities to poor farmers. Understanding the mechanism of CBSD resistance is key for developing disease-resistant cassava plants. To improve our understanding of the mechanisms of CBSV resistance, it is important to identify putative resistance genes that are contributing to disease resistance in cassava plants. Identification of these genes will greatly help the development of gene-targeted molecular markers for breeding that can significantly contribute to the sustainable control of CBSD [1, 11, 12]. This study was aimed at characterizing the molecular responses of CBSV infection in resistant, tolerant, and susceptible cassava varieties. By analysing the transcriptional response to virus infection at molecular pathway levels using several methods, five of virus induced phenylpropanoid pathway genes were identified. To confirm the role of phenylpropanoid pathway genes in CBSD resistance, we performed several complementary experiments; transcriptome analyses of resistant, tolerant and susceptible cassava varieties, induction of PAL genes by ASM application in the susceptible variety, suppression of PAL genes by RNAi in the resistant variety and several gene expression validation assays by qRT-PCR.

Transcriptome analysis of four resistant, two tolerant and two susceptible cassava varieties indicated that a higher number of genes were modulated in tolerant and susceptible varieties up on CBSV infection compared to the resistant varieties. In particular, the resistant var. Kaleso had the lowest number of modulated genes compared to the other seven varieties with varied levels of CBSV resistance. This is similar to the earlier results on cassava [8, 12] and potato [35]. The high gene modulation in tolerant and susceptible varieties might be due to the compatible host–virus interactions. In compatible host–virus interactions the infection by the virus and its multiplication occurs in susceptible and tolerant varieties [8] that triggers a cascade of reactions within the host-plant. Highest number of genes were modulated at early stages of CBSV infection at 1 wai in all cassava varieties compared to the later time points of 5 and 8 weeks, which was also expected as a larger amount of interactions occur between plants and viruses soon after infection than later stages [8, 11, 12]. The gene modulation was either induction or repression depending on the type or family of genes.

RNA-Seq data was analysed to identify candidate genes linked to salicylic acid, jasmonic acid and phenylpropanoid biosynthesis pathways, and no JA and SA hormone signalling pathway genes showed unique induction upon CBSV infection either in resistant or susceptible varieties. Among the signaling pathways, phenylpropanoid pathway genes showed virus-mediated induction in all cassava varieties although the induction was higher in resistant varieties compared to susceptible ones. To correlate the overexpression of phenylpropanoid pathway genes found in RNA-Seq, qPCR analysis was carried out on independent samples of Kaleso and Kalawe plants at 1, 2 and 14 dai, using respective gene-specific primers. The qPCR data showed a strong (5.4 LFC) and early (at 2 dai) induction of phenylpropanoid pathway genes in the resistant Kaleso while substantial repression occurred in the susceptible Kalawe at initial two-time points (2 and 4 dai) although they were induced at late stage of infection (14 dai) and thus representing a slow and delayed response by the susceptible Kalawe. In Kalawe, *C4H* and *CHS* genes were lately induced at 14 dai and *PAL2* was repressed at all time points. We hypothesis that delayed or no induction of PP genes in susceptible KalaweS on CBSV infection might be one of the reasons for its susceptibility. Our results are similar to an earlier study where the resistant var. Kaleso showed high levels of induction of two PAL genes (Manes.15G169500.1 and Manes.12G092100.1) compared to the susceptible var. Albert [11]. Another study [12] also reported the late induction of phenylpropanoid biosynthesis pathways in the CBSD susceptible var. 60,444 at 28 dai compared to the resistant plants. Results from these three independent studies strongly support the involvement of PAL genes in CBSD resistance, where their expression is more rapid, higher, and longer lasting in resistant varieties compared to susceptible ones.

To further confirm the role of *PAL1* in CBSD resistance, elicitor-mediated induction of *PAL1* was performed on cassava plants. This was done to prime the defence system of cassava. Application of ASM induced the expression *PAL1* in both Kaleso and Kalawe plants but the magnitude of expression varied between the cassava varieties and time points. Consistent with the RNA-Seq and qPCR data, ASM treated-Kaleso had highest *PAL1* induction early at 8 hat (0.3 dat) and it lasted until 28 dat. In contrast, unlike RNA-Seq and qPCR data, ASM treated Kalawe plants showed *PAL1* induction at 8 hat and 1 dat but repressed at 2 and 7 dat and induced again at 14 to 28 dat, representing an erratic and inconsistent response to virus infection. Susceptible Kalawe expected to have a compatible interaction with virus (where virus can infect and multiply) and the virus appears to suppress defence gene expression [11]. In this study, ASM application was found modulating/priming the expression of *PAL1* upon CBSV infection in Kalawe plants and effectively delaying and reducing CBSV infection. We also observed that ASM mediated induction of *PAL1* was less in susceptible Kalawe (3.4 fold) compared to Kaleso plants (7.3 fold). Despite the moderate *PAL1* induction, all ASM treated Kalawe plants delayed/suppressed CBSV infection indicating that ASM mediated induction of resistance depended on the type of genotype and their ability to reach their own threshold defence level. Nevertheless, ASM treatment marginally improved the resistance of Kalawe compared to water-treated control plants, and thus further confirming the *PAL1* role in CBSD resistance.

We also carried out transient silencing of *PAL1* by RNAi. Treatment successfully silenced *PAL1* expression (− 13.5 LFC) in the silenced CBSD-resistant Kaleso which led to an increased in virus load at 21 dai, while the control plants were not infected due to the natural expression of phenylpropanoid pathway genes. In summary, our results confirmed that the induction of the *PAL1* gene in the susceptible Kalawe increased its resistance to the disease while its suppression in the resistant Kaleso increased its susceptibility, therefore providing a definitive role for *PAL1* in CBSD resistance. Taken together, our results strongly suggest that early induction of putative resistance genes play an important role in CBSD resistance and *PAL1* is one of the important genes that is contributing to CBSD resistance in cassava. A molecular marker can be developed for *PAL1,* which can be used in future breeding programs to develop new CBSD-resistant cassava varieties. Further studies are required to verify the role of *PAL1* in resistance to UCBSV but we do not expect to be different than CBSV because of high similarity between the viruses and no differences have been reported in cassava varieties resistant to these two viruses.

## Conclusions

Cassava is one of the most important food crops in Africa, providing a staple for > 450 million people in Africa. CBSD is a damaging viral disease of cassava, threatening food security, particularly in eastern, central and southern part of Africa. Transcriptome analysis indicated that host defence responses were highly modulated at early stages of CBSV infection in cassava varieties and CBSD tolerant varieties had highest number of modulated genes. RNA-Seq analysis further indicated the CBSV induced the over expression of phenylpropanoid pathway genes in resistant varieties. qPCR based expression analysis confirmed the strong (up to 41 fold) and early (at 2 dai) induction of phenylpropanoid pathway genes in CBSD-resistant Kaleso and repression or late induction in susceptible Kalawe plants. However, ASM-treated susceptible Kalawe plants showed early induction of *PAL1*, followed by delayed CBSV infection and low virus load, indicating the importance of early induction of *PAL1* in CBSD resistance. In contrast, the RNAi silencing of *PAL1* caused CBSV infection even in the CBSD- resistant Kaleso plants, reaffirming its role in disease resistance. These results strongly suggested that *PAL1* is directly contributing to CBSD resistance in cassava, and early induction of *PAL1* is a key in CBSD resistance.

## Supplementary Information

**Additional file 1**. PAL1 gene of the phenylpropanoid pathway increases resistance to the Cassava brown streak virus in cassava.

## Data Availability

The data that support the findings of this study are available in the Supplementary Information and source data provided with this paper or from the corresponding author upon reasonable request. The RNA-Seq data generated during this study are available at NCBI Sequence Read Archive (SRA) under BioProject PRJNA698085.

## Abbreviations

ASM: Acibenzolar-S-Methyl
CBSD: Cassava brown streak disease
CBSV: *Cassava brown streak virus*
dai: Days after infection
DEGs: Differentially expressed genes
hat: Hours after treatment
Hp RNA: Hair pin RNA
JA: Jasmonic acid
LRR: Leucine-rich repeat
mRNA: Messenger ribonucleic acid
PRGs: Putative resistance genes
RT-qPCR: Reverse transcriptase quantitative PCR
RNAi: Ribonucleic acid interference
RNA-Seq: Ribonucleic acid sequencing
rpm: Revolution per minute(s)
SA: Salicylic acid
