# Supplementary material for: PAL1 gene of the phenylpropanoid pathway increases resistance to the Cassava brown streak virus in cassava
Source: Virol J. 2021 Sep 9;18:184. doi: 10.1186/s12985-021-01649-2 (PMC8428094; doi:10.1186/s12985-021-01649-2)
Supplement: Supplementary file 1 — Additional file 1. PAL1 gene of the phenylpropanoid pathway increases resistance to the Cassava brown streak virus in cassava. [file 12985_2021_1649_MOESM1_ESM.docx]

# **Supplementary Information for The *PAL1* gene of the phenylpropanoid pathway increases resistance to the *Cassava brown streak virus* in cassava**

Siji Kavil, Gerald Otti, Sophie Bouvaine, Andrew Armitage, M. N. Maruthi

M. N. Maruthi

M.N.Maruthi@greenwich.ac.uk

**This file includes:**

Supplementary text

Figures S1 to S4

Tables S1 to S2

Result

**Cassava transcriptome analysis**


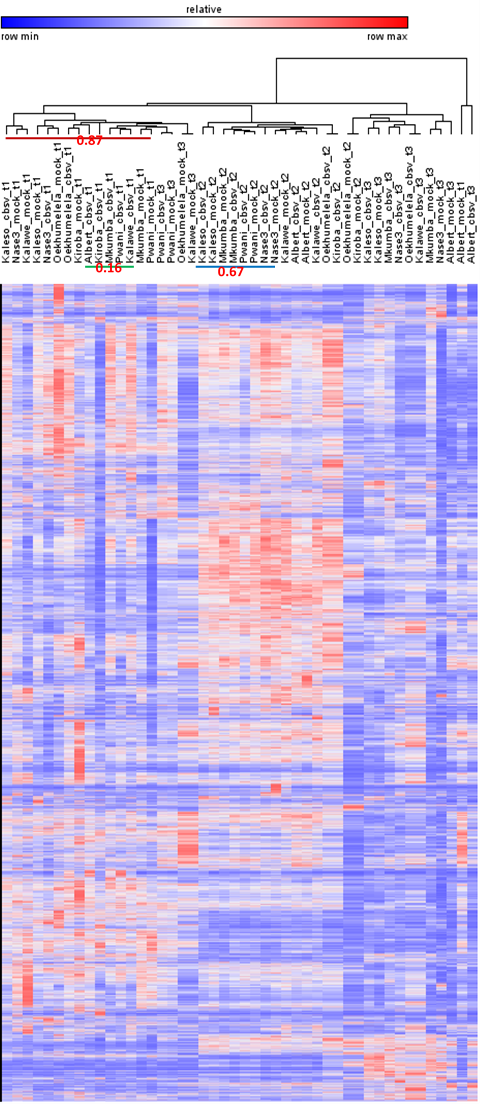


Fig. S1. Heat map of 19,082 gene expression levels in 48 cassava transcriptomes clustered by sample identity and genes. The red, blue and green horizontal bars delineate the longest contiguous sample set under sampling time, infection response category and CBSV treatment-based variety groupings respectively. The figures below these bars represent the respective fraction of samples within variety group contiguous within a cluster.

**Result**

**Genes and signaling pathways enriched in resistant varieties**

Fig. S2. Bar plot of net CBSV-induced expression fold values for gene functional classes averaged across resistant varieties at one, five and eight wai.

**Result**

**Genes and signaling pathways enriched in resistant varieties**

**Fig. S3**. Bar plot of net CBSV-induced expression fold values for gene functional classes averaged across tolerant varieties at one, five and eight wai.

**Result**

**Genes and signaling pathways enriched in resistant varieties**

Fig. S4. Bar plot of net CBSV-induced expression fold values for gene functional classes averaged across tolerant varieties at one, five and eight wai.

Materials and Methods

Phenylpropanoid pathway gene expression

Table S1. List of the primers and probes used in this study

| Primer ID | Tm °C | Gene ID |  | Gene | 5’-3’ sequence | Reference | |
| --- | --- | --- | --- | --- | --- | --- | --- |
| PP2AF | 51.9 | CK650945 |  |  | TGCAAGGCTCACACTTTCATC | 45 |  |
| PP2AR | 55.8 | CK650945 |  |  | CTGAGCGTAAAGCAGGGAAG | 45 |  |
| 591F4 | 58.8 | Manes.04G018000.1 |  | *PAL1* | AAGAGAAGTTCAAGCGCATGTGTG | This study |  |
| 591R4 | 59.1 | Manes.04G018000.1 |  | *PAL1* | TCAAGAGATGGCTCCCAGTAACCG | This study |  |
| 628F2 | 57.2 | Manes.08G008400.1 |  | *PAL2* | AGGAGAACTTGAAGCACGCA | This study |  |
| 628R2 | 56.4 | Manes.08G008400.1 |  | *PAL2* | TGAGCAAGTCCTTCTCGCAG | This study |  |
| 295 F2 | 53.2 | Manes.11G075100.1 |  | CHS | TTCAAGCGCATGTGTGATAAG | This study |  |
| 295 R2 | 55.1 | Manes.11G075100.1 |  | CHS | TGACGAGCATCAAGAGATGG | This study |  |
| 402 F2 | 58.3 | Manes.03G150000.1 |  | CHS | AGTTTCAGCGCATGTGTTGA | This study |  |
| 402 R2 | 57.4 | Manes.03G150000.1 |  | CHS | TGAAGGAGCCATATATGCACAG | This study |  |
| 978 F2 | 54.7 | Manes.18G126900.1 |  | C4H | GTCGCTGCAATTGAAACAAC | This study |  |
| 978 F2R2 | 55.2 | Manes.18G126900.1 |  | C4H | GGTCCAAGCACTGTATCAAG | This study |  |
| RNAi591F | 58.1 | Manes.04G018000.1 |  | *PAL1* | GGGGACAAGTTTGTACAAAAAAGCAGGCTYYTGATTATATGAACAATATGCATGCT | This study |  |
| RNAi591R | 56.8 |  |  | *PAL1* | GGGGACCACTTTGTACAAGAAAGCTGGGTYAGACATTCAAGAAGAGGATCAATGA | This study |  |
| CBSV F3 | 52.0 | FN434437 |  | CBSV | GGARCCRATGTAYAAATTTGC | 35 |  |
| CBSV R4 | 51.8 | FN434437 |  | CBSV | GCWGCQTTTATYACAAAMGC | 35 |  |
| CBSV Probe | 59.6 | FN434437 |  |  | JOE-TTCCAGCCA/ZEN/AGCAATWYTGATGTATCAGAATAGTGTGA-IABkFQ | 31 |  |
| PP2A Probe | 61.3 | CK650945 |  |  | JOE-CTTTCTGTTGCCCCCACCATGC- BHQ-1 | 31 |  |

Materials and Methods

RNAi suppression of *PAL1*

Table S2. RNAi silenced *PAL1* sequences

| TGATTATATGAACAATATGCATGCTTCAATCTTCCTCAAGATAGGTGCTTTCGAAGACGAACTGAAGTCAGTCTTGCCAGAAGAAGTAGAAGCTGCAAGAATTGCTTGTGAGAGTGGGAATCCTGCAGTCCCAAACAGAATCAAAGAGTGCAGGTCTTATCCACTGTACAAGTTCGTGAGAGAAGCTTTGGGGACTGAACTTCTGACAGGAGAGAAGCTGAGGTCTCCTGGTGAGGAATTTGAAAAGTTGTTTACAGCAATTTGTGAAGGAAAGATCATTGATCCTCTTCTTGAATGTCT |
| --- |
